# The Association of Grain Yield and Agronomical Traits with Genes of Plant Height, Photoperiod Sensitivity and Plastid Glutamine Synthetase in Winter Bread Wheat (*Triticum aestivum* L.) Collection

**DOI:** 10.3390/ijms231911402

**Published:** 2022-09-27

**Authors:** Mikhail S. Bazhenov, Ludmila A. Bespalova, Alina A. Kocheshkova, Anastasiya G. Chernook, Olga Y. Puzyrnaya, Elena V. Agaeva, Ekaterina A. Nikitina, Vladimir N. Igonin, Svetlana S. Bazhenova, Elena A. Vertikova, Pyotr N. Kharchenko, Gennady I. Karlov, Mikhail G. Divashuk

**Affiliations:** 1 All-Russia Research Institute of Agricultural Biotechnology, 127550 Moscow, Russia; 2 Department of Breeding and Seed Production of Wheat and Triticale, National Center of Grain Named after P.P. Lukyanenko, Central Estate of KNIISH, 350012 Krasnodar, Russia; 3 Field Experimental Station, Russian State Agrarian University—Moscow Timiryazev Agricultural Academy, 127434 Moscow, Russia; 4 Department of Genetics, Breeding and Seed Production, Russian State Agrarian University—Moscow Timiryazev Agricultural Academy, 127434 Moscow, Russia

**Keywords:** heading date, lodging, molecular markers, nitrogen assimilation, PCR, protein content, semidwarfism

## Abstract

The reduction in plant height caused by mutations in *Rht-B1* or *Rht-D1 (Reduced height-1)* genes in combination with day-length-independent early flowering associated with the *Ppd-D1 (Photoperiod-D1)* gene were the main factors of the drastic yield increase in bread wheat in the 1960s. Increasing nitrogen use efficiency as well as maintaining high yields under conditions of global climate change are the modern goals of wheat breeding. The glutamine synthetase (GS) enzyme plays a key role in ammonium assimilation in plants. In previous studies, the *TaGS2-A1* gene, coding the plastid isoform of GS, was shown to be connected with nitrogen use efficiency in wheat. Using the polymerase chain reaction (PCR) markers, the association of yield and agronomical traits with haplotypes of *Rht-B1*, *Rht-D1*, *Ppd-D1* and *TaGS2-A1* genes was studied in a diverse collection of winter bread wheat cultivars grown in Krasnodar (Russia). In the three-year experiment, semidwarfism and photoperiod insensitivity were confirmed to be highly favorable for the grain yield. The *TaGS2-A1b* haplotype had a tendency for increased grain yield and lodging resistance, but mainly in plants not possessing the ‘green revolution’ alleles. Thus, *TaGS2-A1b* may have potential in breeding wheat cultivars with alternative dwarfing genes or tall cultivars, which may be optimal for growing under certain environments.

## 1. Introduction

Wheat is the staple crop for an estimated 35% of the world’s population [1]. During the “green revolution” of the 1960s, wheat grain yields were nearly doubled due to the introduction of the new short-straw varieties, which better supplied assimilates for the developing spike at the expense of stem biomass and allowed for the application of higher doses of nitrogen fertilizers and irrigation without causing lodging [2,3]. The new varieties were shorter due to limited responsiveness to the growth-promoting gibberellin hormones, which was conferred by mutant alleles at one of the two *Reduced height-1* loci (*Rht-B1b* or *Rht-D1b*) [4]. These genes, located on chromosomes 4B and 4D, correspondingly, encode DELLA proteins, which act in the cell nucleus as regulators of gene transcription. The DELLA proteins consist of a conservative C-terminal domain, responsible for growth repression; and a N-terminal DELLA domain, which is involved in GA perception and targeted degradation of these proteins. The mutations in the DELLA domain of the reduced-height alleles result in the formation of proteins that are not subjected to targeted degradation and thus constitutively repress the growth [5,6]. The height-reducing alleles *Rht-B1b* (synonym of *Rht1*) and *Rht-D1b* (synonym of *Rht2*) are the most widespread, and can be found in modern wheat varieties around the world [7]. Another *Rht-B1e* (synonym of *Rht11*) allele is frequent among Russian cultivars developed in southern regions [8]. The semidwarf allele *Rht-B1p* (synonym of *Rht17*) is promising but not yet commercially used [9], while other alleles of these genes, including superdwarf *Rht-B1c (Rht3)* and *Rht-D1c* (*Rht10*), are not considered to be of commercial value [10,11]. Nonetheless, gibberellin-insensitive reduced-height genes have some adverse effects on agronomic traits, including reduced coleoptile length and seedling vigor [12], lower 1000 grain weight [3,11,13], lower tolerance to drought [4], higher *Fusarium* head blight susceptibility [14] and lower nitrogen use efficiency [15]; they are still widely used in breeding programs. Much research is aimed at alleviating the negative effects of gibberellin-insensitive reduced-height alleles [12,16], while others consider the substitution of these alleles for the gibberellin-sensitive ones [17,18,19].

*Photoperiod-D1* (*Ppd-D1*) is a major photoperiod response locus on chromosome 2D in wheat. It was another gene, along with *Rht-1*, that contributed to the ‘green revolution’. The *Ppd-D1a* allele, which gives the insensitivity to photoperiod, is widespread among Chinese and Japanese landraces [20]. In the early twentieth century, this allele from a Japanese variety ‘Akakomugi’ was passed by Italian breeder Strampelli to European wheat varieties [21]. A semidominant mutation of this locus makes wheat plants photoperiod-insensitive, providing early flowering irrespective of the day length, and thus adapting them to a broad range of environments. Photoperiod neutrality conferred by *Ppd-D1a* gives a substantial yield advantage in Southern Europe and other warm-climate regions, where the season favorable for wheat growing begins under short-day conditions, and earlier flowering allows them to escape the excessive heat during the reproductive phase and the terminal drought stress. In Northern regions, on the contrary, *Ppd-D1a* could be disadvantageous for wheat grain yield [22,23]. *Ppd-D1* is a member of the pseudo-response regulator (PRR) gene family. The photoperiod-insensitive allele *Ppd-D1a* has a 2 kb deletion upstream of the coding region, which is associated with a shifted peak of expression during a day [24] or increased expression [20] of the 2D PRR gene and activation of the key floral regulator *FT* (*Flowering locus T*) even under short-day conditions.

In spite of higher yields, semidwarf ‘green revolution’ varieties were reported to have reduced nitrogen-use efficiency [25]. Reduced-height alleles, associated with accumulation of the DELLA proteins, such as *Rht-B1b*, confer reduced nitrogen growth response and reduced nitrogen uptake both in nitrate and ammonium forms [15]. The *Ppd-D1a* allele may also cause lower nitrogen uptake and use efficiencies [23]. Application of higher doses of nitrogen fertilizers to compensate for this reduction may pose an environmental threat [26]. Thus, for semidwarf wheat varieties, improvement of nitrogen uptake from the soil and nitrogen use efficiency (NUE) seem to be needed.

Better nitrogen uptake and use efficiencies could be conferred by improved root architecture, enhanced work of ammonium and nitrate transporters in cell membranes and higher activity of enzymes of nitrogen metabolism—such as nitrate (NR) and nitrite reductases (NiR), glutamine synthetases (GS) and glutamate synthases (GOGAT), source–sink relationships and senescence dynamics [23].

Glutamine synthetase enzyme (GS) plays a key role in nitrogen utilization, growth and yield potential of cereal crops. It catalyzes a key reaction that incorporates inorganic nitrogen into organic compounds in plants—the synthesis of glutamine (Gln) from glutamate (Glu) and ammonium (NH_4_^+^). Plants have two forms of glutamine synthetase—the cytoplasmic one (GS1) and the plastid one (GS2). The GS1 enzyme is expressed in various organs and tissues and participates in the primary NH_4_^+^ assimilation, as well as in nitrogen remobilization and translocation, while GS2 is localized in chloroplasts and mitochondria of leaves and participates in the assimilation of ammonia formed as a result of nitrite reduction and photorespiration [27,28,29,30]. The GS2 enzyme was shown to be essential for plant survival under photorespiration conditions. During photorespiration, the ammonium is released, while GS2 traps it and incorporates in glutamine, preventing its toxicity and depletion of organic nitrogen in the plant organism [31]. Increased plastid GS expression was shown to be favorable for plant growth and yield as it improves, among other traits, the nitrogen use efficiency. The lines overexpressing GS2 were shown to be more tolerant to abiotic stresses such as drought or soil salinity, as higher GS activity favors the synthesis of osmolytes such as proline or polyamines [31]. Moreover, plants overexpressing GS2 showed increased tolerance to high-intensity light [32].

In bread wheat, the plastid glutamine synthetase (GS2) genes located on chromosomes 2A, 2B and 2D were isolated and sequenced [28]. Two haplotypes were distinguished for *TaGS2-A1* (*a* and *b*), along with six haplotypes for *TaGS2-B1* (*a*–*f*) and two haplotypes for *TaGS2-D1* (*a* and *b*). *TaGS2-A1b* was considered as a favorable one, along with *TaGS2-B1a*, *B1b* and *D1a*, by their association with nitrogen use and agronomic traits in a mini core collection of Chinese bread wheat varieties. In that collection, 65% of accessions possessed *TaGS2-A1b*, and this haplotype was more frequent in modern varieties than in landraces [28]. In another study of durum wheat collection, the variation at *TaGS2-A1* was significantly associated with grain protein content [33]. Expression of the wheat GS2 in *Escherichia coli* showed that an enzyme encoded by the *TaGS2-A1* gene is the most active isoform among others [34].

The transgenic experiment showed that expression of *TaGS2-A1b* under its own promoter in winter bread wheat increases grain yield and its components both under low and high nitrogen supply conditions. In addition, nitrogen acquisition by roots and remobilization was improved in transgenic plants [34]. Thus, *TaGS2-A1b* is valuable in wheat breeding for improved nitrogen use efficiency and grain yield.

In this study, we try to estimate the benefits of the *TaGS2-A1b* haplotype in a mini-collection of winter bread wheat varieties grown in southern Russia and evaluate the prospects of its combined use with well-studied ‘green-revolution’ alleles of *Rht-B1* and *Ppd-D1* genes. We hypothesize that *TaGS2-A1b* can improve nitrogen-use efficiency and further increase grain yields of semidwarf and photoperiod-insensitive wheat plants.

## 2. Results

### 2.1. Sequence Comparison and PCR Marker Development

To investigate the diversity of the *TaGS2-A1* gene using modern genomic information, we extracted the sequences of this gene from 18 wheat accessions having sequenced and assembled genomes, listed in our previous publication [35], most of which are the part of the 10+ Wheat Genomes Project [36]. In the region of the miniature inverted transposable element (MITE) insertion in the second intron, which was previously used as diagnostic for the *TaGS2-A1b* haplotype, some of the genomic sequences were lacking information, and were represented by indefinite nucleotide bases (e.g., ‘NNN’). For further conclusions, we take these regions to be identical to the *TaGS2-A1b* haplotype. Compared to the other sequences, the sequence of the *TaGS2-A1b* haplotype of Xiaoyan 54 (GenBank accession GQ169685.1) has a rare single-nucleotide variant (SNV) within the MITE insertion that differs it from all other genomic sequences having the MITE insertion. If we consider this SNV to be a sequencing error, then only two haplotypes will be found in bread and cultivated durum wheat, which were previously reported as *TaGS2-A1a* (found in Chinese Spring and Lancer bread wheat and in spelt wheat PI 190962) and *TaGS2-A1b* (found in ArinaLrFor, Cadenza, CDC Landmark, CDC Stanley, Claire, Jagger, Julius, Mace, Norin 61, Paragon, Robigus and SY Mattis bread wheat accessions and in durum wheat Kronos). The haplotype *TaGS2-A1b* was obviously prevailing among these accessions of cultivated wheat. In wild emmer wheat Zavitan (*T. turgidum* ssp*. dicoccoides*), two unique SNPs—one in the promotor and another in the sixth intron—were found. The haplotype of Zavitan did not contain the MITE insertion in the second intron. The haplotype of the wild einkorn wheat *Triticum urartu* G1812 (PI428198) contained more unique SNPs and insertions in the promoter and introns, but also did not contain the MITE insertion characteristic for *TaGS2-A1b* (see the supplementary FASTA-format file).

**Figure 1:**
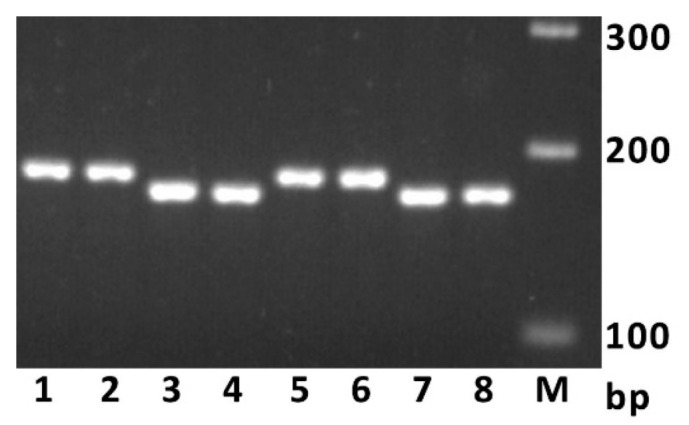
Electrophoresis of the PCR products obtained using primers pGS2-A1-F/R. The lanes represent the following wheat accessions: 1, 2—Caphorn; 3, 4—Nekota; 5, 6—Crimson; 7, 8—Tandem. M—DNA size standard (M-100, Syntol).

*TaGS2-A1b* differs from *TaGS2-A1a* not only by the 239 bp MITE insertion in the second intron, but also by a 10 bp insertion in the 5′ flanking sequence, which obviously serves as a gene promotor. For this 10 bp insertion, we developed the subgenome-specific primers and made a codominant PCR marker suitable for agarose gel electrophoresis. The 172 bp product is diagnostic for haplotype *TaGS2-A1a*, while 182 bp is diagnostic for haplotype *TaGS2-A1b* (Figure 1).

### 2.2. One-Way Analysis

Using this and other molecular markers described in the ‘Materials and Methods’ section, we genotyped the collection of 195 winter bread wheat accessions for *Rht-B1*, *Rht-D1*, *Ppd-D1*, and *TaGS2-A1* genes (Table S13). This collection was grown and evaluated for agronomic traits at the National Center of Grain in Krasnodar (Russia) for three years (2018–2020). Below, we describe the results of genotyping and the associations of the genotypes with the best linear unbiased estimates (BLUEs) of grain yield and agronomic traits for the groups of genes separately and in the triple interaction (*(Rht-B1 + Rht-D1)* × *Ppd-D1* × *TaGS2-A1*).

#### 2.2.1. *Rht-1* Effects

**Figure 2:**
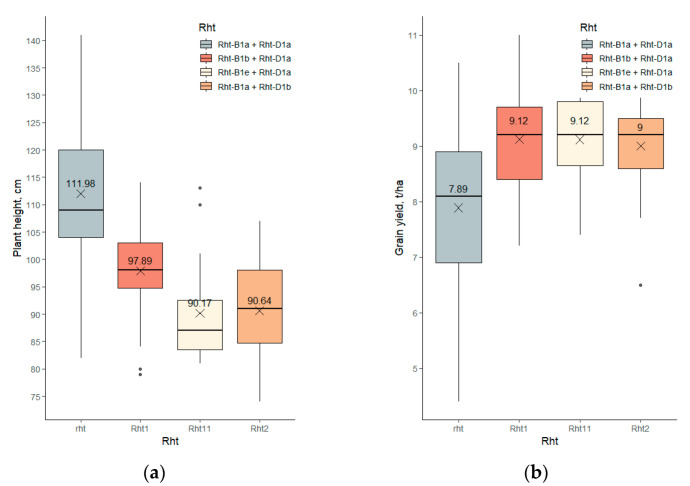
Plant height (**a**) and grain yield (**b**) BLUEs of accessions possessing different alleles of gibberellin-insensitive dwarfing genes in winter bread wheat collection. Here and further on boxplots: the lower and upper boundaries of boxes indicate the first (Q1) and the third (Q3) quartile; the horizontal lines in each box designate the medians; the crosses with the numbers below designate the observed means; the vertical line tips designate the minimum and maximum values within the interval [Q1−1.5(Q3−Q1), Q3+1.5(Q3−Q1)]; the dots indicate outliers.

In 71% of the bread wheat accessions, one of the three gibberellin-insensitive reduced-height alleles was found: *Rht-B1b* was found in 47%, *Rht-D1b* was found in 14%, and *Rht-B1e* was found in 9% of accessions. In 29% of accessions, none of these reduced-height alleles were detected (Table S13). In addition, there were no double dwarfs combining *Rht-D1b* with *Rht-B1b* or *Rht-B1e* in the collection studied. The effects of these reduced-height alleles on plant height were slightly different: The accessions carrying *Rht-D1b* or *Rht-B1e* had a lower average plant height (91 ± 3 and 90 ± 5 cm, respectively) than those carrying *Rht-B1b* (98 ± 1 cm; here and further the means of BLUEs with the 95% confidence intervals are given). The average height of accessions without gibberellin-insensitive reduced-height alleles was 112 ± 3 cm (Figure 2a). The effects of the three *Rht* alleles were also a bit different for the other agronomical traits. Accessions carrying *Rht-D1b* had a reduced 1000-kernel weight, but at the same time they were the most resistant to lodging, substantially differing in this feature from all other genotypes. The grain yield per hectare of the three gibberellin-insensitive reduced-height genotypes did not differ significantly from each other (Figure 2b), but their average yield (9.1 ± 0.1 t/ha) was significantly higher than that of ‘tall’ accessions (7.9 ± 0.3 t/ha). The grain protein content was decreased by semidwarfism; however, the grain protein yield per hectare was increased. Unexpectedly, the lodging tolerance of accessions carrying the gibberellin insensitivity alleles was not substantially higher (Table S1).

#### 2.2.2. *Ppd-D1* Effects

The allele *Ppd-D1a*, conferring photoperiod insensitivity, was observed in 76% of wheat accessions; 19% of accessions had the *Ppd-D1b*; the remaining 5% of accessions were heterogenous (Table S13). Only the homogenous accessions were used for calculating any effects on agronomical traits. On average, accessions with *Ppd-D1a* proceeded to heading 7 days earlier than accessions with *Ppd-D1b* (Table S2). In addition, *Ppd-D1a* increased 1000-kernel weight by 3.8 g. The grain yield in photoperiod-insensitive accessions was higher by 0.7 t/ha. *Ppd-D1a* decreased the grain protein content by 0.7%; however, the grain protein yield slightly increased by 0.5 t/ha. In photoperiod-insensitive plants carrying *Ppd-D1a*, the leaf area damaged by brown rust (*Puccinia triticina* f. sp. *tritici*) was around twice lower. Despite a little reduction of the plant height, the *Ppd-D1a* allele noticeably decreased the lodging tolerance of the wheat plants.

#### 2.2.3. *GS2-A1* Effects

Using the developed marker, the *TaGS2-A1b* haplotype, previously considered as more favorable for nitrogen assimilation, was detected in 43% of accessions, while *TaGS2-A1a* was observed in 53% of them. A total of 4% of accessions were heterogenous for *TaGS2-A1.* By the results of one-way ANOVA, *TaGS2-A1* did not influence any of the traits significantly. However, there was a tendency for *TaGS2-A1b* to increase lodging resistance and to delay heading.

### 2.3. Multifactor Analysis

In the studied collection, the wheat accessions that combine any of the reduced-height *Rht-1* alleles with the allele of insensitivity to the photoperiod *Ppd-D1a* are prevailing. Since both the reduced-height and photoperiod insensitivity genes have a strong effect on many agronomical traits, and the combinations of alleles of these genes in the collection are not strictly random (slightly more accessions combining insensitivity to photoperiod with gibberellin-insensitive dwarfism, *p* = 0.02 for χ^2^), it is better to view the effects of these genes in interaction, based on the results of multivariate analysis. Since, in general, the effects of various gibberellin-insensitive *Rht* alleles have the same direction, to simplify the interpretation of the results we decided to unite the data of three gibberellin-insensitive alleles markers to divide the collection in two groups: Those with such alleles (*Rht-B1b*, *Rht-B1e*, *Rht–D1b*) will be called ‘dwarf’, and those without them will be called ‘tall’. Further, the results of the three-factor analyses (*(Rht-B1 + Rht-D1)* × *Ppd-D1* × *TaGS2-A1*) accounting for the gene interactions are presented.

#### 2.3.1. Heading Date

**Figure 3:**
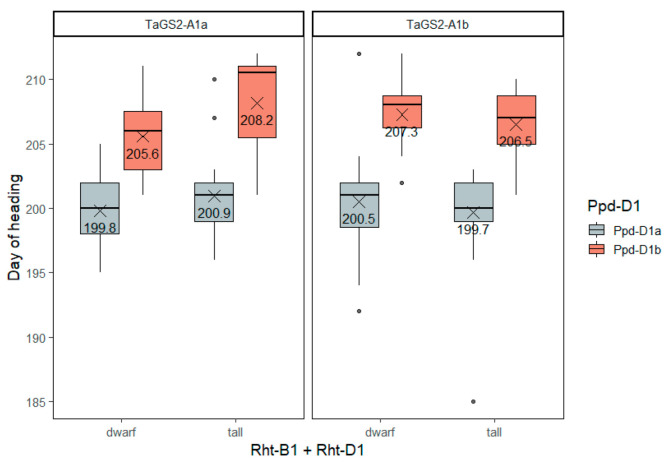
The heading date (days from sowing) of winter wheat accessions differing in genotypes of the three groups of genes—*Rht-B1 + Rht-D1*, *Ppd-D1*, *TaGS2-A1*. The so-called ‘tall’ accessions are those that have both *Rht-B1a* and *Rht-D1a* wild-type alleles, while ‘dwarf’ ones are those possessing either *Rht-B1b*, *Rht-B1e* or *Rht-D1b* alleles. The dots indicate outliers.

As expected, *Ppd-D1* explained most of the heading date genetic variance in the winter bread wheat collection (Table S4). Accounting for the Bonferroni correction, other factors did not significantly influence the heading date. The accessions carrying *Ppd-D1a*, the allele conferring photoperiod insensitivity, headed 6 to 7 days earlier than accessions with *Ppd-D1b* (Table S12, Figure 3).

#### 2.3.2. Plant Height

**Figure 4:**
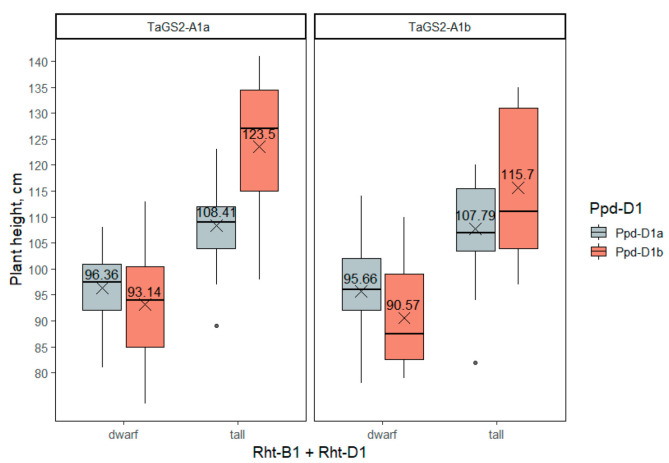
The plant height of wheat accessions differing in genotypes of the three groups of genes—*Rht-B1 + Rht-D1*, *Ppd-D1*, *TaGS2-A1*. The dots indicate outliers.

Most part of the plant height variance was explained by gibberellin-insensitive dwarfing genes *Rht-B1* and *Rht-D1*, and their interaction with *Ppd-D1* (Table S5). As expected, the height of the wheat plants carrying one of the gibberellin-insensitivity alleles was from 12 to 30 cm lower depending on the genetic background. The photoperiod-insensitive allele *Ppd-D1a* also reduced the height of plants by 8–15 cm, but only in accessions not carrying the *Rht-B1* or *Rht-D1* dwarfing alleles (Figure 4, Table S12). Variation at the *TaGS2-A1* locus did not have a significant impact on plant height, neither by itself nor in interaction with *Rht-1* or *Ppd-D1*.

#### 2.3.3. Grain Yield

**Figure 5:**
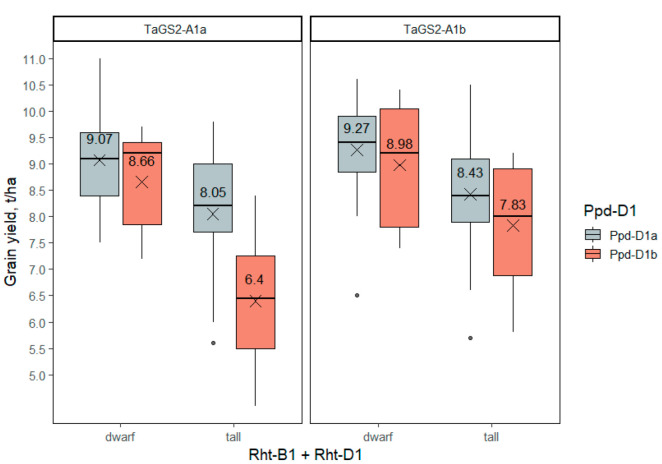
The grain yield of wheat accessions differing in genotypes of the three groups of genes—*Rht-B1 + Rht-D1*, *Ppd-D1*, *TaGS2-A1*. The dots indicate outliers.

The yield of grain (t/ha) was significantly affected by the *Rht-1* and *Ppd-D1* genes, while *TaGS2-A1* had a marginally significant effect if the interaction of the genes had been accounted (Table S6). The semidwarf, insensitive to photoperiod accessions, as well as those carrying *TaGS2-A1b*, showed higher three-year BLUEs of grain yield than those carrying wild-type alleles. On average, semidwarfism increased the grain yield by 1.4 t/ha, insensitivity to photoperiod increased the grain yield by 0.8 t/ha and *TaGS2-A1b* increased the grain yield by 0.6 t/ha. Tall, long-day accessions carrying *TaGS2-A1a* had the lowest grain yield (6.4 ± 0.8 t/ha), significantly differing from other genotypes. The presence of at least one of the favorable alleles of *Rht-1*, *Ppd-D1* or *TaGS2-A1* increased the yield substantially. Further yield increase due to the combination of favorable alleles of the two or three genes in one genotype was not so strong, but statistically significant. The highest grain yield, 9.3 ± 0.3 t/ha, was observed in accessions combining “positive” alleles of all three genes (*Rht-B1b* or *Rht-B1e* or *Rht-D1b* together with *Ppd-D1a* and *TaGS2-A1b*) (Figure 5, Table S12).

#### 2.3.4. Leaf Rust

**Figure 6:**
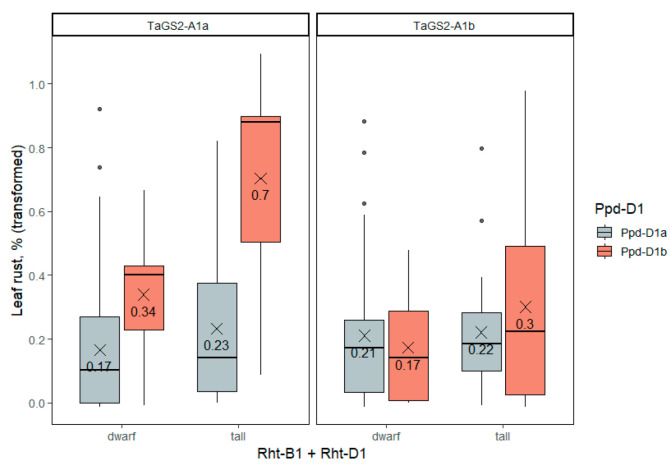
Leaf area damaged by brown rust in winter wheat accessions differing in *Rht-1*, *Ppd-D1* and *TaGS2-A1* genotypes, proportion transformed with $arcsin(\sqrt{x})$. The dots indicate outliers.

The genotype of the *Ppd-D1* gene showed near-significant association with the percentage of leaf area damaged by brown rust (Table S7). Wheat accessions having the allele of photoperiod neutrality, *Ppd-D1a*, were less affected by leaf rust compared to long-day plants. On average, the percentage of leaf area affected by leaf rust was 5% in day-length-neutral accessions and 12% in day-length-sensitive accessions. The genes *TaGS2-A1* and *Rht-1* and genetic factor interactions showed no significant effects on this trait in the three-factor analysis. For the factor analysis, the near-absolutely resistant accessions were excluded for data to fit the normal distribution. These near-absolutely resistant accessions were more frequent among those that carried the *TaGS2-A1b* allele, as we can see from Figure 6, where all the data were used.

#### 2.3.5. The 1000-Kernel Weight

**Figure 7:**
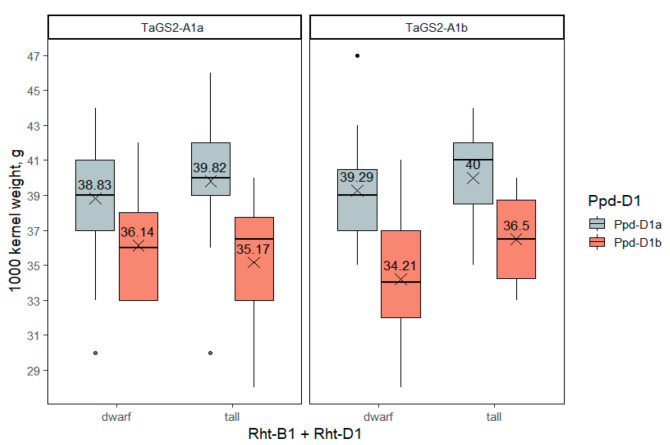
The 1000-kernel weight in wheat accessions differing in *Rht-1*, *Ppd-D1* and *TaGS2-A1* genotypes. The dots indicate outliers.

The 1000-kernel weight was significantly affected only by the *Ppd-D1* gene (Table S8). In photoperiod-insensitive accessions, the 1000-kernel weight averaged 39.2 ± 0.5 g, while in long-day accessions it was 35.4 ± 1.0 g. The *Rht-1* and *TaGS2-A1* genes did not influence 1000-kernel weight significantly (Figure 7).

#### 2.3.6. Grain Protein Content

**Figure 8:**
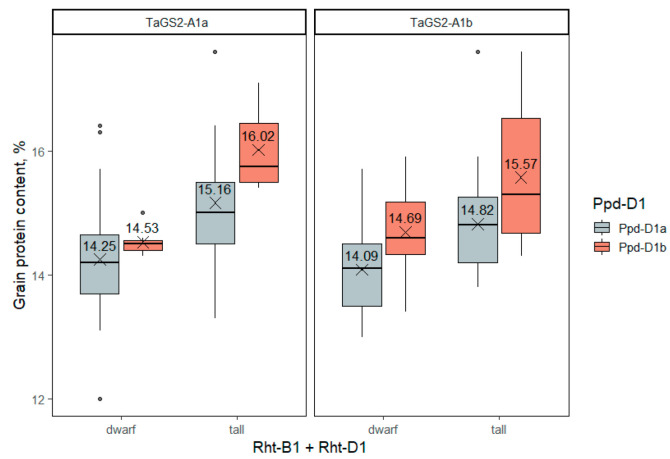
Grain protein content (%) in winter wheat accessions differing in *Rht-1*, *Ppd-D1* and *TaGS2-A1* genotypes. The dots indicate outliers.

Both *Rht-1* and *Ppd-D1* genes showed a significant effect on the grain protein content (Table S9). In both semidwarf and photoperiod-neutral forms, the protein content was significantly lower (Figure 8). The gibberellin-insensitive semidwarf alleles reduced the grain protein content by about 1% (from 15.4 to 14.4%), while day-length insensitivity reduced it by about 0.6% (from 15.2 to 14.6%). The *TaGS2-A1* gene, however, did not significantly affect the protein content. In addition, there was no statistically significant interaction of the three genetic factors, which indicates their additive effect on the trait.

#### 2.3.7. Grain Protein Yield

**Figure 9:**
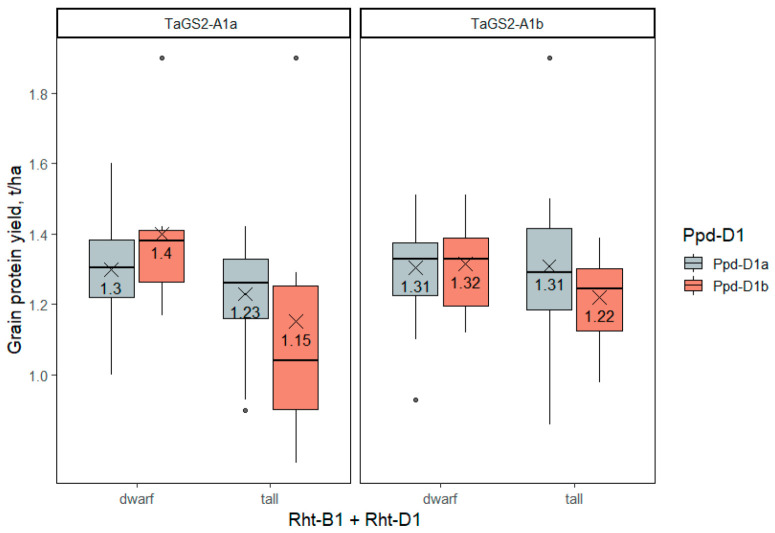
Grain protein yield (t/ha) in winter wheat accessions differing in *Rht-1*, *Ppd-D1* and *TaGS2-A1* genotypes. The dots indicate outliers.

The grain protein yield per hectare was significantly influenced by *Rht* genes, and was marginally significant by the *TaGS2-A1* gene (Table S10). Despite the decrease in protein content, the grain protein yield per hectare was significantly higher in semidwarf accessions compared to tall ones. The reason for that was an overcompensating increase in grain yield. The presence of one of the reduced-height alleles (*Rht-B1b*, *RhtB1e* or *Rht-D1b*) gave an increase in protein yield by 0.13 t/ha (from 1.16 to 1.29 t/ha), and the presence of the *TaGS2-A1b* allele increased the protein yield by 0.08 t/ha (from 1.18 to 1.26 t/ha). The lowest protein yield, significantly different from other genotypes, was observed in ‘tall’ accessions carrying *Ppd-D1b* and *TaGS2-A1a* alleles (Figure 9, Table S12).

#### 2.3.8. Lodging Resistance

**Figure 10:**
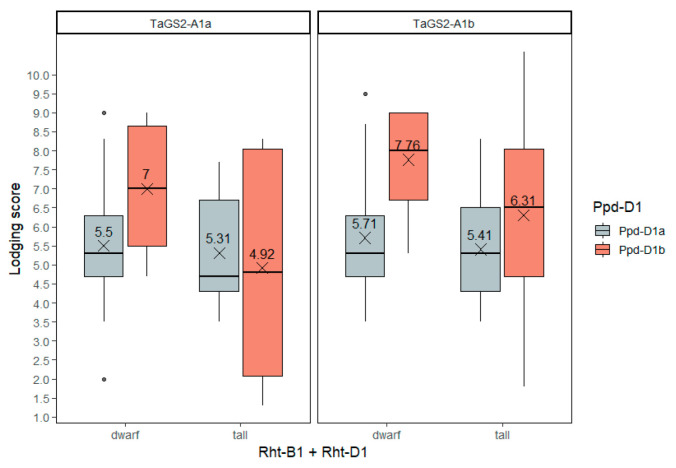
Lodging score BLUEs (9—no lodging, 2—total lodging) for winter wheat genotypes. The dots indicate outliers.

As expected, semidwarfism caused by the *Rht-B1* and *Rht-D1* dwarfing alleles was connected with higher resistance to lodging (higher values of the lodging score) (Table S11). Insensitivity to photoperiod caused by the *Ppd-D1a* allele, despite decreased plant height, was accompanied by lower values of lodging resistance. The *TaGS2-A1b* allele had a tendency to improve lodging resistance (Figure 10, Table S12). The most lodging-resistant accessions were semidwarf, photoperiod-sensitive and those carrying the *TaGS2-A1b* allele.

## 3. Discussion

The studied collection of winter bread wheat accessions tested in Krasnodar consists of old and new cultivars and advanced breeding lines. In our study, about 70% of the collection possessed one of the reduced-height gibberellin-insensitive alleles of either *Rht-B1* or *Rht-D1* genes, and the most frequent of them was *Rht-B1b*. These alleles provide optimal semidwarf plant height and about 15% higher grain yield. This shows that *Rht-B1b*, *Rht-B1e* and *Rht-D1b* dwarfing alleles are still current for winter wheat breeding in Russia, despite known poor adaptability of gibberellin-insensitive dwarf cultivars to dry environments. The climate of Krasnodar is characterized by prolonged, dry and hot summers. However, the winter wheat growing season spans autumn; winter, which in Krasnodar is relatively short and mild, without constant snow cover; and spring, which is usually favorable for plant growth. Thus, winter crops can escape the adverse conditions of heat and drought. Nevertheless, more efforts are being conducted by breeders recently to shorten the vegetation period of winter wheat. On the other hand, our results show that rather high yields could be achieved without gibberellin-insensitive dwarfism.

We also noted that reduced-height alleles, obviously having the same mechanism of action, cause various degrees of agronomical trait changes. *Rht-D1b* or *Rht-B1e* cause stronger height reduction than *Rht-B1b*, although the grain yield is not much different between groups of accessions having one or another allele of these three. This is consistent with previous findings [3,8].

Generally, the presence of gibberellin-insensitive reduced-height alleles in our study was associated with reduced grain protein content. That is consistent with other studies, and could be explained by the ‘dilution’ of grain protein by a larger amount of stored carbohydrates [37]. However, grain protein yield per hectare is substantially higher in accessions carrying one of these dwarfing alleles than in ‘tall’ genotypes. Considering that all accessions in the field experiments were grown under equal conditions, this contradicts the states of some articles claiming weak nitrogen use efficiency in gibberellin-insensitive semidwarf cultivars [15].

*Ppd-D1a*, the photoperiod-insensitive allele, was observed in 76% of accessions in our collection, which is a relatively high proportion, showing its adaptability for winter wheat in warmer regions. Under conditions of southern Russia, *Ppd-D1a* gives improvement for several agronomic traits, including earlier heading date, lower plant height and higher 1000-grain weight. Above all, *Ppd-D1a* is favorable for higher grain yield per hectare. All these *Ppd-D1* effects were reported in previous studies [10]. Shortening the vegetation period obviously allows them to escape the terminal drought and heat stresses, and it will be even more actual in the future, as according to the prognoses, global warming will be accompanied by shrinkage of autumn, winter and spring [38].

Despite mildly decreased plant height, the photoperiod-insensitive accessions carrying *Ppd-D1a* were less tolerant to lodging. That could be caused by the greater spike mass of these plants, which bends the stems to the ground. However, weaker stems due to faster development rate could be a reason. Further research should be conducted to elucidate this fact.

The leaf rust-affected area was significantly lower in accessions carrying *Ppd-D1a*. In our study, the leaf rust was scored 10 days after heading for each accession individually. Thus, a higher rate of development could allow the photoperiod-insensitive plants to escape the peak of disease development in the field. Previously, the connection of photoperiod insensitivity with disease resistance was reported in wheat, and was explained mainly by the shift of the development stages against the weather events [39]. Alternatively, we can assume co-occurrence of horizontal resistance genes with *Ppd-D1a* in the same cultivars due to breeding. The race-nonspecific resistance genes were widely introduced in breeding programs together with genes of reduced height and insensitivity to photoperiod, and co-occurrence of them in wheat cultivars of diverse origin could be expected [40].

The *TaGS2-A1* gene encodes the most active isoform of plastid glutamine synthase in hexaploid wheat, which performs one of the key steps of nitrogen assimilation. Previously, two haplotypes were discovered (*TaGS2-A1a* and *TaGS2-A1b*) differing mainly within noncoding sequences and promotors, and two synonymous SNPs in protein-coding sequences. Previous studies showed advantage of the *TaGS2-A1b* haplotype in hexaploid wheat [28].

In our study, the *TaGS2-A1b* haplotype was weakly positively connected with higher grain yield, grain protein yield (but not grain protein content) and lodging resistance. The highest effects were observed within accessions that do not carry any of the ‘green revolution’ alleles of *Ppd-D1*, *Rht-B1* or *Rht-D1* genes. This is consistent with the study of the *TaGS2-A1* gene in the Chinese wheat collection, where *TaGS2-A1b* showed positive effects on plant biomass under any nitrogen supply only among landraces, but not in bred cultivars (most of which are supposed to possess some of the ‘green revolution’ alleles, while landraces are not). Thus, we can hypothesize that the *TaGS2-A1b* haplotype could be effective, if one needs to refuse gibberellin-insensitive dwarfism. For example, in many regions of Central Asia, where drought begins earlier during vegetative growth and anthesis, taller genotypes perform much better than semidwarf ones [4]. On the other hand, it may be of value in more northern nonchernozem regions, where *Ppd-D1a* is not as favorable as in Krasnodar Krai.

A connection of glutamine synthetase with lodging was not expected, but as we know, lodging resistance of wheat depends not only on plant height, but also on the strength of the culm, anchorage power of the root system, and the weight of the spike [41]. Improved nitrogen assimilation in plants carrying *TaGS2-A1b* could result in the development of stronger stems, less prone to lodging.

Further studies should be aimed at glutamine synthetase biochemical activity, stem anatomy and disease immunity in wheat lines with various genotypes of *Rht*, *Ppd* and *TaGS2* genes to elucidate our findings.

## 4. Materials and Methods

### 4.1. Plant Material and Phenotyping

The winter bread wheat (*Triticum aestivum* L.) accessions used in this study were a part of the collection of the National Center of Grain named after P.P. Lukyanenko in Krasnodar, Russia. The origin of accessions and references for their brief characteristics were published in our previous study [35].

Yield testing and phenotyping for agronomical traits were conducted during 2018–2020 harvest years in Krasnodar. The methodology of field experiments and weather conditions during growing seasons were published in our previous study [35]. The brown rust (*Puccinia triticina f. sp. tritici*) was scored 10 days after heading as a visual estimation of the percentage of the leaf area occupied by the disease.

### 4.2. STS Marker Development for the TaGS2-A1 Gene

The sequences of the glutamine synthetase gene *TaGS2-A1* were obtained from the NCBI database (accession numbers GQ169684, GQ169685) [28], and further from the Chinese Spring wheat genome IWGSC RefSeq v1.0 [42], and from the sequences of the 10+ Wheat Genomes Project [36] using BLAST+ software [43] as described earlier [35]. The sequences were aligned and compared using GeneDoc2.7 software [44].

The gene-specific pair of primers flanking the 10 bp insertion/deletion in the promotor (pGS2-A1-F/pGS2-A1-R, Table 1) was designed using Primer-BLAST (NCBI) [45]. The specificity of the primers was checked using alignment of the three homoeologous genes of GS2 (TraesCS2A02G500400, TraesCS2B02G528300, TraesCS2D02G500600).

The PCR for the *TaGS2-A1* STS (sequence-tagged site) marker was performed in 25 μL reaction volumes, containing 1× buffer solution supplied in a kit with the polymerase (70 mM Tris–HCl, pH 8.6, 16.6 mM (NH_4_)_2_SO_4_, 2.5 mM MgCl_2_ in final volume; Sileks Ltd., Moscow, Russia), 0.2 mM of each dNTP (Sintol Ltd., Moscow, Russia), 0.3 μM forward and reverse primers (Sintol Ltd., Moscow, Russia), 0.05 U/µL Taq polymerase (Sileks Ltd., Moscow, Russia) and 4 ng/µL DNA template. The PCR conditions were as follows: (1) 95 °C for 10 min, (2) 36 cycles of 95 °C for 30 s, 60 °C for 30 s, 72 °C for 1 min; and (3) final extension step of 72 °C for 10 min. PCR products were separated in 2% agarose gels with TBE buffer for at least 1 h in an electric field intensity of 6 V/cm, stained with ethidium bromide and documented under UV light using the Gel Doc XR+ system (Bio-Rad Laboratories, Inc., Hercules, CA, USA).

### 4.3. DNA Extraction and PCR-Markers

Genomic DNA was extracted from ground dried leaves of seedlings using the CTAB-based protocol [46]. The DNA samples of the two individual plants from each wheat accession were used for genotyping.

Detection of the dwarfing *Rht-B1b*, *Rht-B1e* (TraesCS4B02G043100) and *Rht-D1b* (TraesCS4D02G040400) alleles was performed using allele-specific polymerase chain reaction (ASPCR) markers [47]. For these markers, usually two separate PCRs are performed, and in each mixture a common primer and allele-specific primer with 3′ end complementary to the SNP detected are used. The PCR products of each reaction are detected by gel electrophoresis on distinct lanes, and then results are combined to tell homozygotes for one or another allele from heterozygotes. Two pairs of primers were used for detection of *Rht-B1b*: BF + MR1 for finding the mutant allele, and BF + WR1 for the wild-type allele. *Rht-D1b* was detected using primers DF + MR2, and *Rht-D1a* using DF2 + WR2 primers. For *Rht-B1e*, two primer pairs were used: BF with WR3 to amplify wild-type sequences, while BF with MR3 to amplify a fragment only for *Rht-B1e* allele [48].

Detection of *Ppd-D1* (TraesCS2D02G079600) alleles was performed using a PCR with 3 primers: common primers Ppd-D1_F and Ppd-D1_R1 for wild-type allele *Ppd-D1b*, giving a 414 bp product; and Ppd-D1_R2 for the mutant ‘photoperiod-insensitive’ allele *Ppd-D1a* with the deletion in a promotor of the gene, giving a 288 bp product [24].

The sequences of the primers used for PCR are represented in Table 1**.** The PCR conditions were used as described in the original protocols. The PCR products were separated in 1.5% agarose gel with TBE buffer for 30 min at electric field intensity of 6 V/cm, stained with ethidium bromide and documented under ultraviolet light using the Gel Doc XR+ system.

### 4.4. Statistical Analysis

The statistical distribution of the majority of the traits tested resembled the normal distribution, except for the values of the leaf rust damage. Before further processing, the percentages of the leaf rust damage were transformed using $arcsin(\sqrt{x})$, as this transformation keeps the zero values. The best linear unbiased estimates (BLUEs) of the three years of measurements were calculated using Tassel 5 software [49]. The BLUEs for the leaf rust were back-transformed to percentages to be presented in Table S13. To obtain the best fit for the normal distribution, the near-to-zero (<0.2%) BLUE values of the leaf rust damage were discarded (as absolute resistance is not assumed to be phenotype for the genes tested), and the remaining values were *ln(x)-*transformed. The BLUEs were used for the analysis of variance regarding the molecular markers in Statistica 6.0. We used one-way analysis of variance (ANOVA) for separate genes or a group of *Rht-1* genes united through assumed phenotype (tall or semidwarf) as a single factor and separate traits as dependent variables. In addition, we used factorial ANOVA to estimate the gene interaction and to compensate for the allele imbalance in the wheat collection. Presence of GA-insensitive dwarfing alleles (1), alleles of *Ppd-D1* (2) and *TaGS2-A1* (3) were treated as factors. The factor analysis included each of the three factors (1), (2), (3), their double interactions (1 × 2), (1 × 3), (2 × 3) and the triple interaction (1 × 2 × 3) as fixed effects. Sigma-restricted parameterization and type VI (unique) sum of squares were used. Fisher’s F-test was used to estimate the significance of the effects. The adjusted α levels were calculated using the Bonferroni–Holm correction for 8 comparisons (8 traits) in one-way ANOVA and 56 comparisons (8 traits × 7 factors, including double and triple interactions) for multifactorial ANOVA [50]. The least-square means were calculated as an estimation of the group means; the differences between means were accessed using Tukey’s HSD (honestly significant difference) test. The boxplots for 3-year BLUEs were built using Rstudio 2021.09.1 Build 372 software, R 4.1.2 programming language and ggplot2 package [51].

## 5. Conclusions

Semidwarfism associated with *Rht-B1* or *Rht-D1* mutations and earliness connected to the *Ppd-D1a* allele are highly beneficial for the grain yield of winter bread wheat in the southern regions of Russia. The accessions carrying *Rht-B1b*, *Rht-B1e* or *Rht-D1b* lightly differ in plant height but not in the grain yield per unit area. Semidwarfism caused by these alleles was associated with lower grain protein content, but with higher protein yield per unit of area. Despite a mild decrease in plant height provided by the allele of photoperiod insensitivity *Ppd-D1a*, the accessions carrying it are less resistant to lodging. The *TaGS2-A1b* haplotype of the plastid glutamine synthetase gene has a tendency to improve lodging resistance and grain yield. However, in the presence of ‘green revolution’ alleles, the positive effects of *TaGS2-A1b* on agronomical traits are minimal. Thus, *TaGS2-A1b* may have potential in breeding taller wheat cultivars or cultivars with alternative dwarfing genes, which may perform better under drier environments.

## Figures and Tables

**Table 1:** The primers used for the PCR markers.

Name	Sequence (5′->3′)	Alleles Detected, Product Length
BF	GGTAGGGAGGCGAGAGGCGAG	(Used with MR1, WR1, MR3, WR3)
MR1	CATCCCCATGGCCATCTCGAGCTA	*Rht-B1b*, 237 bp
WR1	CATCCCCATGGCCATCTCGAGCTG	*Rht-B1a* (not *Rht-B1b*) *, 237 bp
MR3	GGCCATCTCCAGCTGCTCCAGCTA	*Rht-B1e*, 228 bp
WR3	GGCCATCTCCAGCTGCTCCAGCTT	*Rht-B1a* (not *Rht-B1e*) *, 228 bp
DF	CGCGCAATTATTGGCCAGAGATAG	*Rht-D1b*, 254 bp
MR2	CCCCATGGCCATCTCGAGCTGCTA	
DF2	GGCAAGCAAAAGCTTCGCG	*Rht-D1a* (not *Rht-D1b*) *, 264 bp
WR2	GGCCATCTCGAGCTGCAC	
Ppd-D1_F	ACGCCTCCCACTACACTG	*Ppd-D1a*, 288 bp; *Ppd-D1b*, 414 bp
Ppd-D1_R1	GTTGGTTCAAACAGAGAGC	
Ppd-D1_R2	CACTGGTGGTAGCTGAGATT	
pGS2-A1-F	GGCCTCCGCTCCCATAAATATAA	*TaGS2-A1a*, 172 bp; *TaGS2-A1b*, 182 bp
pGS2-A1-R	AACGCAACAGAGATTGAAGAAGC	

* The nucleotide identical to wild-type allele at SNP is detected.

## Data Availability

The data presented in this study are available in Supplementary Materials to this article.

## Supplementary Materials

The following supporting information can be downloaded at: https://www.mdpi.com/article/10.3390/ijms231911402/s1, Tables S1–S13.
