# Supplementary material for: The Association of Grain Yield and Agronomical Traits with Genes of Plant Height, Photoperiod Sensitivity and Plastid Glutamine Synthetase in Winter Bread Wheat (Triticum aestivum L.) Collection
Source: Int J Mol Sci. 2022 Sep 27;23(19):11402. doi: 10.3390/ijms231911402 (PMC9570164; doi:10.3390/ijms231911402)
Supplement: Supplementary file 1 [file ijms-23-11402-s001.zip › Supplementary_materials/Tables S1-S12.pdf]

Supplementary materials

Table S1. Aggregated gibberellin-insensitive dwarfing genes (*Rht-B1* + *Rht-D1*) effects on plant traits

Trait	Means ± 95% CI		p-value
	**'dwarf'**	**'tall'**	
Heading date (days after sowing)	201±1	202±1	0.077
Plant height, cm	95±2	112±3	0.000*
Grain yield, t/ha	9.1±0.2	7.9±0.3	0.000*
Leaf rust area fraction, transformed[1]	-3.0±0.3	-2.5±0.4	0.078
1000 kernel weight, g	38.2±0.6	38.7±0.9	0.338
Grain protein content, %	14.3±0.1	15.2±0.2	0.000*
Grain protein yield, t/ha	1.30±0.02	1.19±0.04	0.000*
Lodging score	5.9±0.3	5.4±0.4	0.064

CI – the confidence intervals. The p-values lower than 0.006 (0.05/8) are marked with an asterisk.
[1] The $ln(x)$ transformation for leaf area fractions damaged by brown rust (*Puccinia triticina* f. sp. *tritici*) was applied before statistical analysis, the fractions below 0.002 (0.2%) were excluded from analysis.

Table S2. *Ppd-D1*gene effects on plant traits

Trait	Means ± 95% CI		p-value
	Ppd-D1a	***Ppd-D1b***	
Heading date (days after sowing)	200±0.5	207±1.0	0.000*
Plant height, cm	99±2	103±4	0.067
Grain yield, t/ha	8.9±0.2	8.2±0.3	0.000*
Leaf rust area fraction, transformed	-3.0±0.3	-2.1±0.5	0.004*
1000 kernel weight, g	39.2±0.5	35.4±1.0	0.000*
Grain protein content, %	14.4±0.1	15.1±0.3	0.000*
Grain protein yield, t/ha	1.28±0.02	1.23±0.04	0.037
Lodging score	5.5±0.3	6.8±0.5	0.000*

Table S3. *TaGS2-A1* gene effects on plant traits

Trait	Means ± 95% CI		p-value
	TaGS2-A1a	***TaGS2-A1b***	
Heading date (days after sowing)	201±1	202±1	0.012
Plant height, cm	100±2	100±3	0.964
Grain yield, t/ha	8.7±0.2	8.8±0.2	0.318
Leaf rust area fraction, transformed	-2.8±0.3	-2.9±0.4	0.601
1000 kernel weight, g	38.6±0.7	38.0±0.7	0.252
Grain protein content, %	14.5±0.2	14.6±0.2	0.904
Grain protein yield, t/ha	1.25±0.03	1.28±0.03	0.229
Lodging score	5.5±0.3	6.1±0.4	0.025

Table S4. Heading date BLUEs analysis of variance

Effect	SS	df	MS	F	p
Rht-B1+Rht-D1	6	1	6	0.6	0.438
Ppd-D1	1112	1	1112	119.5	0.000*
TaGS2-A1	0	1	0	0.1	0.823
(Rht-B1+Rht-D1)×Ppd-D1	3	1	3	0.3	0.596
(Rht-B1+Rht-D1)×TaGS2-A1	45	1	45	4.8	0.029
Ppd-D1×TaGS2-A1	0	1	0	0.0	0.853
(Rht-B1+Rht-D1)×Ppd-D1×TaGS2-A1	4	1	4	0.4	0.527
Error	1600	172	9		

Here and in tables S5 – S11: SS – the sum of squares, df – the degrees of freedom, MS – the mean square (the variance), F – the F-test of equality of variances statistic, p – the p-value. * The p-values below the adjusted α level calculated with the Bonferroni–Holm correction for 56 comparisons (7 factors × 8 traits) are marked with an asterisk.

Table S5. Plant height BLUEs analysis of variance

Effect	SS	df	MS	F	p
Rht-B1+Rht-D1	9971	1	9971	122.4	0.000*
Ppd-D1	333	1	333	4.1	0.045
TaGS2-A1	195	1	195	2.4	0.124
(Rht-B1+Rht-D1)×Ppd-D1	1547	1	1547	19.0	0.000*
(Rht-B1+Rht-D1)×TaGS2-A1	35	1	35	0.4	0.512
Ppd-D1×TaGS2-A1	121	1	121	1.5	0.225
(Rht-B1+Rht-D1)×Ppd-D1×TaGS2-A1	43	1	43	0.5	0.468
Error	14014	172	81		

Table S6. Grain yield BLUEs analysis of variance

Effect	SS	df	MS	F	p
Rht-B1+Rht-D1	44.2	1	44.2	48.5	0.000*
Ppd-D1	13.6	1	13.6	14.9	0.000*
TaGS2-A1	8.7	1	8.7	9.6	0.002
(Rht-B1+Rht-D1)×Ppd-D1	3.7	1	3.7	4.0	0.046
(Rht-B1+Rht-D1)×TaGS2-A1	2.7	1	2.7	3.0	0.086
Ppd-D1×TaGS2-A1	2.3	1	2.3	2.5	0.115
(Rht-B1+Rht-D1)×Ppd-D1×TaGS2-A1	1.4	1	1.4	1.5	0.220
Error	156.6	172	0.9		

Table S7. Leaf area fraction[1] damaged by brown rust BLUEs analysis of variance

Effect	SS	df	MS	F	p
Rht-B1+Rht-D1	2.84	1	2.84	1.454	0.230
Ppd-D1	14.41	1	14.41	7.387	0.008
TaGS2-A1	3.35	1	3.35	1.720	0.192
(Rht-B1+Rht-D1)×Ppd-D1	0.67	1	0.67	0.345	0.558
(Rht-B1+Rht-D1)×TaGS2-A1	0.90	1	0.90	0.464	0.497
Ppd-D1×TaGS2-A1	1.30	1	1.30	0.667	0.416
(Rht-B1+Rht-D1)×Ppd-D1×TaGS2-A1	0.22	1	0.22	0.113	0.738
Error	220.42	113	1.95		

[1]The $ln(x)$ transformation was applied to the fractions of the leaf area before ANOVA

Table S8. 1000 kernel weight BLUEs analysis of variance

Effect	SS	df	MS	F	p
Rht-B1+Rht-D1	18.0	1	18.0	2.03	0.156
Ppd-D1	384.4	1	384.4	43.31	0.000*
TaGS2-A1	0.0	1	0.0	0.00	0.955
(Rht-B1+Rht-D1)×Ppd-D1	0.1	1	0.1	0.01	0.924
(Rht-B1+Rht-D1)×TaGS2-A1	15.9	1	15.9	1.80	0.182
Ppd-D1×TaGS2-A1	1.9	1	1.9	0.21	0.648
(Rht-B1+Rht-D1)×Ppd-D1×TaGS2-A1	19.6	1	19.6	2.21	0.139
Error	1526.7	172	8.9		

Table S9. Grain protein content % BLUEs analysis of variance

Effect	SS	df	MS	F	p
Rht-B1+Rht-D1	26.03	1	26.03	42.79	0.000*
Ppd-D1	9.77	1	9.77	16.07	0.000*
TaGS2-A1	0.92	1	0.92	1.52	0.220
(Rht-B1+Rht-D1)×Ppd-D1	0.90	1	0.90	1.49	0.224
(Rht-B1+Rht-D1)×TaGS2-A1	1.05	1	1.05	1.73	0.190
Ppd-D1×TaGS2-A1	0.08	1	0.08	0.14	0.713
(Rht-B1+Rht-D1)×Ppd-D1×TaGS2-A1	0.28	1	0.28	0.46	0.497
Error	104.60	172	0.61		

Table S10. Grain protein yield (t/ha) BLUEs analysis of variance

Effect	SS	df	MS	F	p
Rht-B1+Rht-D1	0.367	1	0.367	21.43	0.000*
Ppd-D1	0.101	1	0.101	5.91	0.016
TaGS2-A1	0.134	1	0.134	7.83	0.006
(Rht-B1+Rht-D1)×Ppd-D1	0.064	1	0.064	3.76	0.054
(Rht-B1+Rht-D1)×TaGS2-A1	0.037	1	0.037	2.17	0.142
Ppd-D1×TaGS2-A1	0.069	1	0.069	4.04	0.046
(Rht-B1+Rht-D1)×Ppd-D1×TaGS2-A1	0.025	1	0.025	1.44	0.232
Error	2.949	172	0.017		

Table S11. Lodging score BLUEs analysis of variance

Effect	SS	df	MS	F	p
Rht-B1+Rht-D1	24.8	1	24.8	10.5	0.001*
Ppd-D1	26.4	1	26.4	11.1	0.001*
TaGS2-A1	9.5	1	9.5	4.0	0.048
(Rht-B1+Rht-D1)×Ppd-D1	14.1	1	14.1	5.9	0.016
(Rht-B1+Rht-D1)×TaGS2-A1	0.4	1	0.4	0.1	0.700
Ppd-D1×TaGS2-A1	5.2	1	5.2	2.2	0.140
(Rht-B1+Rht-D1)×Ppd-D1×TaGS2-A1	0.8	1	0.8	0.3	0.565
Error	408.8	172	2.4		

Table S12. The estimated marginal means of plant traits for genotypes calculated in triple gene interaction

Genotype			Day of heading	Plant height, cm	Grain yield, t/ha	1000 kernel weight, g	Grain protein content, %	Grain protein yield, t/ha	Leaf rust area fraction, *ln*(*x*) transformed	Lodging score
Rht-B1+Rht-D1	***Ppd-D1***	***TaGS2-A1***								
**dwarf**	***Ppd-D1a***	***TaGS2-A1a***	200±1a	96±2a	9.1±0.2cd	39±1bc	14.2±0.2ab	1.29±0.03b	-3.1±0.4a	5.5±0.4a
**dwarf**	***Ppd-D1a***	***TaGS2-A1b***	201±1a	96±3a	9.3±0.3d	39±1bc	14.1±0.3a	1.31±0.04b	-3.0±0.6a	5.7±0.5a
**dwarf**	***Ppd-D1b***	***TaGS2-A1a***	206±2b	93±7a	8.7±0.7bcd	36±2abc	14.5±0.6abc	1.26±0.10b	-2.1±1.0a	7.0±1.2ab
**dwarf**	***Ppd-D1b***	***TaGS2-A1b***	207±2b	91±5a	9.0±0.5bcd	34±2a	14.7±0.4abc	1.31±0.07b	-2.7±0.8a	7.8±0.8b
**tall**	***Ppd-D1a***	***TaGS2-A1a***	201±1a	108±4b	8.0±0.5b	40±1c	15.2±0.4cd	1.21±0.06b	-2.6±1.0a	5.3±0.7a
**tall**	***Ppd-D1a***	***TaGS2-A1b***	200±1a	108±4b	8.4±0.4bc	40±1c	14.8±0.4bc	1.24±0.06b	-3.1±0.7a	5.4±0.7a
**tall**	***Ppd-D1b***	***TaGS2-A1a***	208±2b	123±7c	6.4±0.8a	35±2ab	16.0±0.6d	1.02±0.11a	-1.4±1.9a	5.0±1.2a
**tall**	***Ppd-D1b***	***TaGS2-A1b***	206±2b	116±6bc	7.8±0.6ab	37±2abc	15.6±0.5cd	1.21±0.08ab	-2.2±1.3a	6.3±1.0ab

The 95% confidence intervals are given. The letters designate the homogenous groups determined using the Tukey's range test, α= 0.05. For lodging score 9 – no lodging, 2 – total lodging.
